# Harnessing the diversity of wild emmer wheat for genetic improvement of durum wheat

**DOI:** 10.1007/s00122-022-04062-7

**Published:** 2022-03-07

**Authors:** Mohammed Yousif Balla, Yasir Serag Alnor Gorafi, Nasrein Mohamed Kamal, Modather Galal Abdeldaim Abdalla, Izzat Sidahmed Ali Tahir, Hisashi Tsujimoto

**Affiliations:** 1 United Graduate School of Agricultural Sciences, Tottori University, Tottori, 680-8553 Japan; 2 Arid Land Research Center, Tottori University, 1390 Hamasaka, Tottori, 680-0001 Japan; 3 Agricultural Research Corporation, P.O. Box 126, Wad Medani, Sudan

## Abstract

**Key message:**

The multiple derivative lines (MDLs) characterized in this study offer a promising strategy for harnessing the diversity of wild emmer wheat for durum and bread wheat improvement.

**Abstract:**

Crop domestication has diminished genetic diversity and reduced phenotypic plasticity and adaptation. Exploring the adaptive capacity of wild progenitors offer promising opportunities to improve crops. We developed a population of 178 BC_1_F_6_ durum wheat (*Triticum turgidum* ssp*. durum*) lines by crossing and backcrossing nine wild emmer wheat (*T. turgidum* ssp*. dicoccoides*) accessions with the common durum wheat cultivar ‘Miki 3’. Here, we describe the development of this population, which we named as multiple derivative lines (MDLs), and demonstrated its suitability for durum wheat breeding. We genotyped the MDL population, the parents, and 43 Sudanese durum wheat cultivars on a Diversity Array Technology sequencing platform. We evaluated days to heading and plant height in Dongola (Sudan) and in Tottori (Japan). The physical map length of the MDL population was 9 939 Mb with an average of 1.4 SNP/Mb. The MDL population had greater diversity than the Sudanese cultivars. We found high gene exchange between the nine wild emmer accessions and the MDL population, indicating that the MDL captured most of the diversity in the wild emmer accessions. Genome-wide association analysis identified three loci for days to heading on chromosomes 1A and 5A in Dongola and one on chromosome 3B in Tottori. For plant height, common genomic loci were found on chromosomes 4A and 4B in both locations, and one genomic locus on chromosome 7B was found only in Dongola. The results revealed that the MDLs are an effective strategy towards harnessing wild emmer wheat diversity for wheat genetic improvement.

**Supplementary Information:**

The online version contains supplementary material available at 10.1007/s00122-022-04062-7.

## Introduction

Durum wheat (*Triticum turgidum* ssp. *durum*, genome BBAA) is a tetraploid wheat species (2*n* = 28), mainly used for human consumption in the form of pasta, bulgur, couscous, and some bread types (Al-Khayri et al. 2019). In terms of consumption and area under cultivation, durum wheat ranks second after bread wheat with 5% of the total cultivated wheat area (Mastrangelo and Cattivelli 2021). Although the Mediterranean region accounts for—about half of the total world durum wheat production, it still remains the largest importer and consumer of durum wheat (Royo et al. 2021). Durum wheat, which evolved from wild emmer wheat (WEW: *Triticum turgidum* ssp. *dicoccoides*), shares two of the three sub-genomes with common wheat (*T. aestivum*). The best accepted scenario for its evolution involved two successive domestication events; with the first event leading to the replacement of the brittle type of WEW with a non-brittle type, which produced the first domesticated forms of emmer wheat (*T. turgidum* ssp*. dicoccum*)*.* The second event accounted for the further domestication of emmer forms into the modern free-threshing durum wheat (*T. turgidum* ssp. *durum*) (Gioia et al. 2015; Kabbaj et al. 2017; Maccaferri et al. 2019). During these two events, population sizes reduced, resulting in limited genetic diversity, now explained as bottlenecks (Lopes et al. 2015). In durum wheat, 84% of the nucleotide diversity in WEW has been lost during the domestication events (Haudry et al. 2007). Furthermore, Maccaferri et al. (2019) reported that a great reduction in diversity occurred during recent breeding activities. The loss of genetic diversity in modern durum wheat cultivars restricts the improvement of durum wheat for high productivity and increases its vulnerability to biotic and abiotic stresses (Abdurakhmonov and Abdukarimov 2008). This narrow genetic diversity hinders the identification of efficient QTLs and genes necessary for its genetic improvement. However, despite the impressive results achieved during the Green Revolution (Borlaug 2007), major equity problems encountered by the farmers, stability, and sustainability led to calls for a ‘new phase’ of agricultural research and development (Conway and Barbie 1988). The Green Revolution impacted negatively on sustainable agriculture through the increased use of fertilizers and mono-cropping systems, decreasing soil quality and reducing crop biodiversity (Eliazer Nelson et al. 2019). In addition, we now face the urgent need of doubling productivity for a projected human population of 9.2 × 10^9^ by 2050 (Rasheed et al. 2018). Hence, breeders have adopted the application of genomics, phenotyping technologies, and analytical tools in breeding diverse populations to maximize opportunities for better selection towards the improvement of staple food crops such as wheat. To this end, wheat breeders now focus on enriching the gene pool by reintroducing valuable wild alleles that were changed, modified, lost, or left behind during the domestication process (Tsujimoto et al. 2015; Merchuk-Ovnat et al. 2016; Gorafi et al. 2018). The diversity in WEW needs to be explored as it played a central role in the domestication of durum wheat.

WEW is considered as a source of valuable genetic diversity that offers important agronomic, biotic, and abiotic stress-related traits (Matsuoka 2011; Peng et al. 2011; Rahman et al. 2020). The similarity of the WEW genomes to the durum and a part of bread wheat facilitates the transfer of any gene of interest into cultivated wheats. WEW has been shown to have two lineages of diversity which could be exploited for wheat improvement via genetic introgression: the western lineage, colonizing Israel, Syria, Lebanon, and Jordan and central-eastern lineage, dominating in Turkey, Iraq, and Iran (Mori 2003; Ozkan et al. 2005; Matsuoka 2011; Peng et al. 2011).

Substantial research efforts have been devoted to developing diverse durum wheat populations to exploit the WEW diversity via the advantage of genomic tools (Peleg et al. 2008, 2009; Avni et al. 2014; Merchuk-Ovnat et al. 2016; Jorgensen et al. 2017). However, this effort needs the integration of representatives of the two lineages of WEW diversity in one population.

Using such a strategy, we developed a population harboring the diversity of the two WEW lineages by crossing and backcrossing nine *T. turgidum* ssp. *dicoccoides* accessions with the common durum wheat cultivar ‘Miki 3’. Collectively, we named the lines in this population multiple derivative lines (MDLs). This work describes the MDL development strategy, its suitability and potential for durum and bread wheat genetic improvement, genome-wide association (GWA) analysis, and gene mining from WEW. This population is available to the wheat community upon request from the Laboratory of Arid Land Plant Resources of the Arid Land Research Center of Tottori University, Japan.

## Materials and methods

### Plant materials

We used nine WEW accessions provided by the National BioResource Project—Wheat, at Kyoto University; durum wheat (*T. turgidum* ssp. *durum*) cultivar ‘Miki 3’ provided by Dr. M. Nachit, International Center for Agricultural Research in the Dry Areas (ICARDA); and 43 elite Sudanese durum wheat lines. The MDL population was bred from ‘Miki 3’ and nine wild emmer accessions, namely KU-108-1, KU-108-4, and KU-108-5, of unknown origin (UN); KU-8808, KU-8810, KU-8814, and KU-8815, from Iraq (IQ); and KU-14474 and KU-14532, from Israel (IL) (Table 1). ‘Miki 3’ was chosen because it is a leading durum wheat cultivar in some Mediterranean countries including Lebanon and Syria where it is known as ‘Berdawni’ and ‘Cham 7’, respectively, and has high yield, high resilience in irrigated environments, and resistance to yellow rust and leaf rust (Afifi and Sastry 2013). Sudanese cultivars included five released cultivars (‘Cham 1’, ‘Zaidab’, ‘Argu’, ‘Basatna’, and ‘Wadelbur’) and 38 advanced lines as checks.

**Table 1:** Accessions of wild emmer wheat, *Triticum turgidum* ssp. *dicoccoides* used in this study and their origin

Code	Accession number	Origin
1	KU-108–1	Unknown (UN)
2	KU-108–4	Unknown (UN)
3	KU-108–5	Unknown (UN)
4	KU-8808	Iraq (IQ)
5	KU-8810	Iraq (IQ)
6	KU-8814	Iraq (IQ)
7	KU-8815	Iraq (IQ)
8	KU-14474	Israel (IL)
9	KU-14532	Israel (IL)

### Production of the MDL population

First, we crossed the nine WEW accessions as males with ‘Miki 3’ to produce nine F_1_ hybrids in 2011. In 2012, we backcrossed nine F_1_ plants as females with ‘Miki 3’ and obtained nine BC_1_F_1_ families consisting of 236 plants. Ten self-pollinated seeds from each of 10 BC_1_F_1_ plants in the nine families were mixed and planted as a population of 900 BC_1_F_2_ plants. Of the 900 plants, 369 (41%) showed hybrid necrosis and died. We harvested bulked seeds from the remaining 531 plants and named this population Multiple Derivative Lines (MDLs) BC_1_F_2_. All plants showed diverse morphology; 453 plants had a waxy stem and 78 were waxyless. In 2014, we sowed the seeds from the 531 bulked plants and obtained BC_1_F_3_ MDLs from 466 surviving plants. In 2015, we randomly selected 1000 seeds from the BC_1_F_3_ MDLs and produced BC_1_F_4_ MDLs. All these activities were conducted at the Arid Land Research Center, Tottori University. In 2016, based on the agronomically desired traits (heading, non-shattering, and free-threshing) we selected and evaluated 501 plants from the 1000 BC_1_F_4_ population at the Agricultural Research Corporation (ARC), Wad Medani, Sudan, as separate lines and selected 225 potential lines. In 2018, we re-evaluated the 225 selected BC_1_F_5_ lines at Wad Medani and selected 178 BC_1_F_6_ with good agronomic performance (Supplementary Fig. 1). These 178 MDLs were used as a validation panel for the MDL platform.

## DNA extraction, DArTseq genotyping, and genetic analysis

Total genomic DNA was isolated using the CTAB method (Saghai-Maroof et al. 1984), and DNA samples (20 µL; 50–100 ng µL^−1^) were sent to Diversity Array Technology (DArT) Pty. Ltd., Australia (http://www.diversityarrays.com), for whole-genome scanning with DArTseq (DArT sequencing) markers. Restriction fragments from each sample were sequenced and aligned to durum wheat cv. ‘Svevo’ RefSeq v. 1.0 (Maccaferri et al. 2019).

Pedigree analysis, principal component analysis (PCA), phylogenetic analysis, estimation of Nei’s genetic diversity index, and analysis of molecular variance (AMOVA) were conducted in Flapjack v. 1.20.10.07 (Milne et al. 2010), R v.4.0.3, PowerMarker v. 3.25 (Liu and Muse 2005), MEGA X (Kumar et al. 2018), and GenAlex v. 6.5 software (Peakall and Smouse 2012), respectively. In the pedigree analysis, we chose each of the nine WEW accessions as the first parent and ‘Miki 3’ as the second parent, and then selected each of the MDL progeny with ≥ 75% similarity to the first parent as putative progeny. From AMOVA, the pairwise population (Phipt) and Nm (haploid number of migrants) within the population were obtained from GenAlexv.65. Marker deviation from the expected Mendelian segregation ratio of 3:1 was evaluated by using the following equation:

$$\chi = - (O_{{\text{w}}} - 0.25n)/0.25n + (O_{{\text{m}}} - 0.75n)/0.75n$$

where *n* is total number of lines in a family, and *O* is observed number of lines with a (w) WEW or (m) ‘Miki 3’ allele in the family. The χ value is the deviation from the expected ratio; a large value indicates deviation to ‘Miki 3’ and a small value to WEW.

MDL individuals were clustered with a discriminant analysis of principal component (DAPC) implemented in R/adegenet (Jombart et al. 2010) to identify genetic similarity between MDL families. A clustering algorithm based on Bayesian information criteria (BIC) was used to determine the number of clusters in the MDLs.

### Phenotypic evaluation of the MDL population

To test the usefulness and suitability of the MDL population for durum wheat breeding and genome-wide association analysis, we measured days to heading (DH) and plant height (PHT) in Dongola (Sudan) and in Tottori (Japan) because these traits are extensively studied in durum wheat. In both locations, DH was measured as the number of days from the first irrigation or transplanting until 50% of the plant headed. PHT was recorded at maturity by measuring the distance between the ground and the top spike excluding awns.

In Sudan, the 178 MDLs and ‘Miki 3’ were grown during the winter season (2019–2020) at Dongola Research Station Farm (19°08′ N, 30°27′ E, and 239 m a.s.l.), Agricultural Research Corporation (ARC), Sudan. The soil is high terrace soil (pH 8.0 − 8.4) with low organic matter content < 5% (Elbashir et al. 2017). Seeds were dressed with insecticide/fungicide mixture of Gaucho (Imidacloprid 39% WP, Bayer Crop Science, Kansas City, MO, USA) at 0.75 g kg^−1^. Sowing was performed manually at the rate of 120 kg ha^−1^ during the 1st week of December. Fertilization was done using DAP (Diammonium phosphate) or triple superphosphate by furrow placement before planting at the rate of 43 kg ha^−1^ of P_2_O_5_, whereas urea was split-applied by broadcasting before the second and fourth irrigation at the rate of 86 kg ha^−1^. In this location, there is no rainfall during the wheat growing season, and irrigation system was carried out at 10–14 days intervals following the ARC recommendation (wheat water requirement is about 400 mm) to avoid water stress. Weeding was done manually at least twice in both locations. All the cultural practices were conducted according to the ARC recommendations for wheat production. The average minimum and maximum temperatures during the season were 11.4 °C and 28.3 °C, respectively.

In Japan, seeds of the genetic materials were germinated on tray pots and transferred to the field of the Arid Land Research Center (35°32′ N, 134°13′ E, 11 a.s.l.), Tottori in the second week of December and harvested in mid-June. This location has a high cold winter with rain-fed field conditions, and the average minimum and maximum temperatures during the season were 7.1 and 16.2 °C, respectively, and the rainfall amount was 930 mm (Arid Land Research Center weather station). The field description and management were the same as described by Elhadi et al. (2021).

Each field experiment was arranged in an alpha-lattice design with two replicates. The plot size was four rows, 1 m long, 0.2 m apart in Dongola, and one row with five plants 0.2 m apart in Tottori.

### Genome-wide association (GWA) analysis

We conducted GWA analysis with the genotyping data (DArTseq markers) and phenotypic data. We used a mixed linear model (MLM) incorporating the population structure as fixed effect and kinship matrix as random effect among the individuals, in TASSEL v. 5.2.66 software (Bradbury et al. 2007). In total, 13 312 SNPs markers with a call rate of 90% (10% missing data) and MAF (minor allele frequency) > 0.05 were used in the analysis. The threshold of *P* < 0.0001 (− log_10_ (*P*) > 4) indicated the degree of association between each SNP marker and a trait, and *R*^2^ was the variation explained by the significantly associated markers. The MLM product from TASSEL was used in R v. 4.0.3 with custom scripts in the developed GWAS package rMVP to draw Manhattan plots and quantile–quantile plots (Yin et al. 2021).

## Results

### Genotyping of the MDL population

**Fig. 1:**
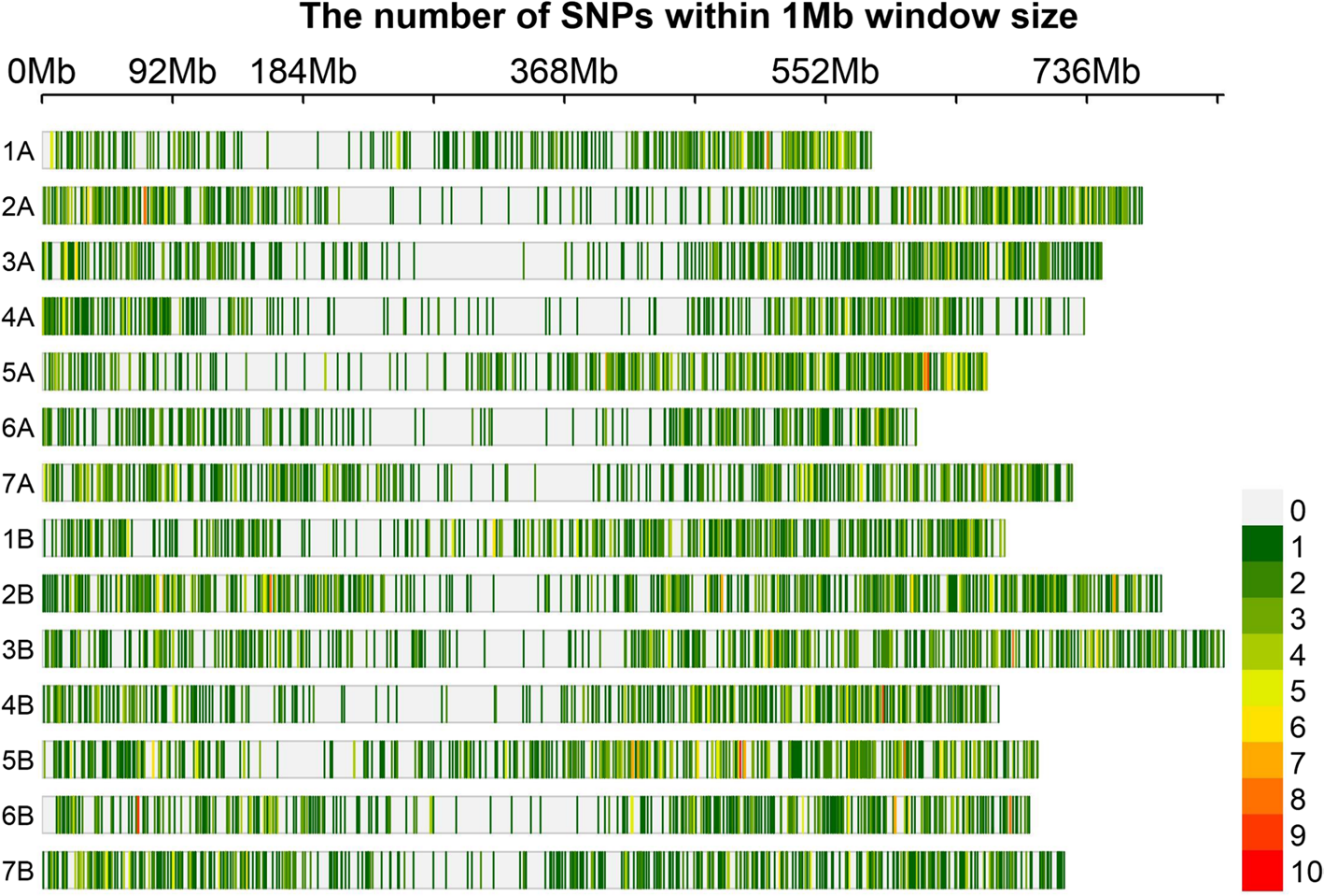
Distribution of SNPs of 7275 DArTseq markers among 178 multiple derivative lines, nine wild emmer wheat accessions, and 43 Sudanese durum wheat cultivars. Redder markers have higher density; greener markers have lower density; gray areas have no markers (color figure online)

The DArTseq genotyping platform provides two types of markers: Silico-DArT markers (SiD), scored as presence or absence, and SNP markers. We obtained 54 712 SiD and 64 817 SNP markers. The genetic positions of 628 SiD and 7 275 SNP markers with a call rate of 100% (no missing data) were determined on the 14 durum wheat chromosomes (Fig. 1). As SNP markers are codominant and are used widely, we used them for most of our analysis with no missing data. By SNP genotyping, the total length of the physical map was 9 939 Mb. The longest chromosomes were 3B (832 Mb) and 2B (788 Mb), and the shortest was 1A (583 Mb). The average physical distance between SNPs was 1.4 Mb. The length of the A genome was 4 845 Mb and B genome 5 088 Mb. Chromosome (Chr) 2B had the most SNPs (715), and 6A had the fewest (318). The SNP markers were denser in the telomeric regions than in the centromeric regions.

### Comparison of geographical origin and genetic relationship between population parents and Sudanese cultivars

**Fig. 2:**
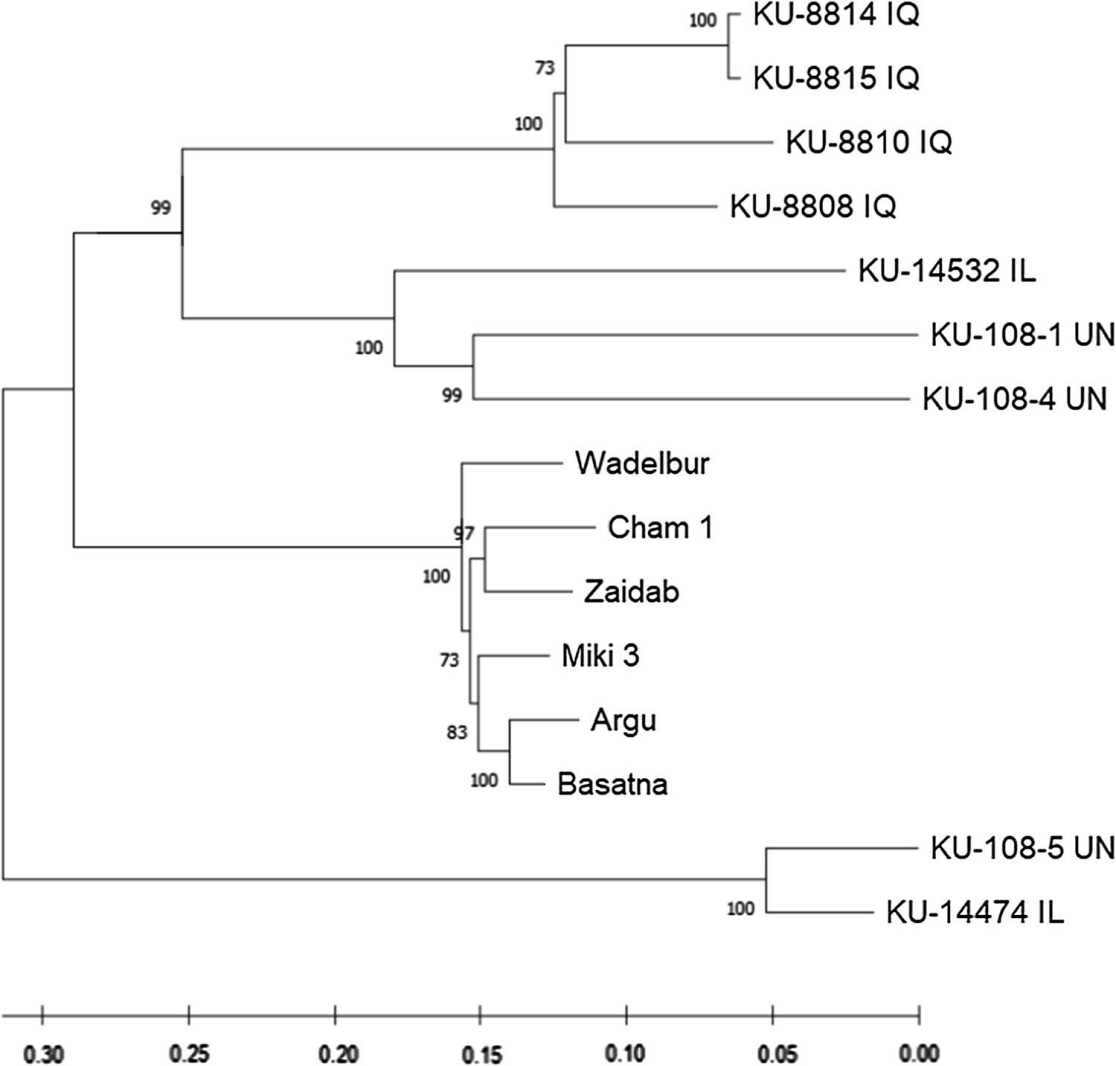
Phylogenetic analysis to elucidate the genetic relationship between the Sudanese cultivars and parents used to generate the multiple derivative lines. The recurrent parent ‘Miki 3’ is a durum wheat cultivar; the other nine parents (with code KU) are wild emmer wheat (WEW) accessions, country abbreviation code IL indicate Israel, IQ indicate Iraq and UN indicate unknown origin. Sudanese cultivars are five released cultivars (‘Cham 1’, ‘Zaidab’, ‘Argu’, ‘Basatna’, and ‘Wadelbur’). All Sudanese cultivars and ‘Miki 3’ are clustered in one group, whereas the nine WEW are placed together in another groups

Phylogenetic analysis using 7 275 SNPs markers placed the Sudanese cultivars and ‘Miki 3’ in one group and the nine WEW accessions in another group (Fig. 2). The latter was further divided into two sub-groups, one with an accession from Israel and one of unknown origin, and the other with the remaining seven accessions. This latter group was further divided into two sub-groups, one with an accession from Israel and two of unknown origin, and the other with four accessions from Iraq (Fig. 2). Accessions KU-8814 and KU-8815 from Iraq have substantial genetic similarity. The passport data revealed that they are derived from the same line, so they are probably separated because of their distinct characteristics.

### Pedigree of the MDL lines

**Fig. 3:**
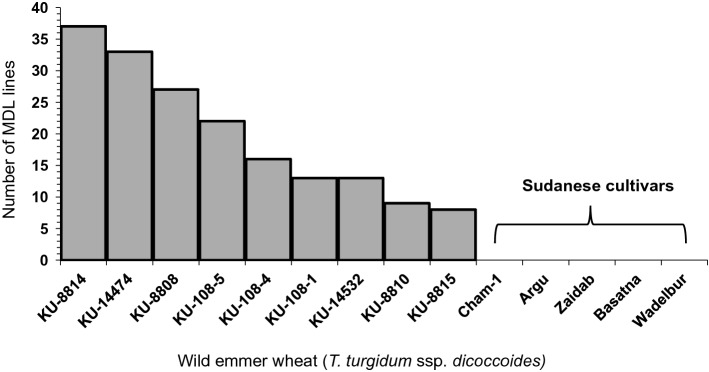
Number of progenies from each of the nine wild emmer wheat accessions (range, 7–37). Five Sudanese cultivars were used as checks

Since each of 178 MDLs comes from bulked population, we sought the pedigree of these lines. Each WEW accession produced from 7 to 37 MDLs (Fig. 3). In contrast, there was no similarity of the five Sudanese cultivars to the nine WEW accessions.

**Fig. 4:**
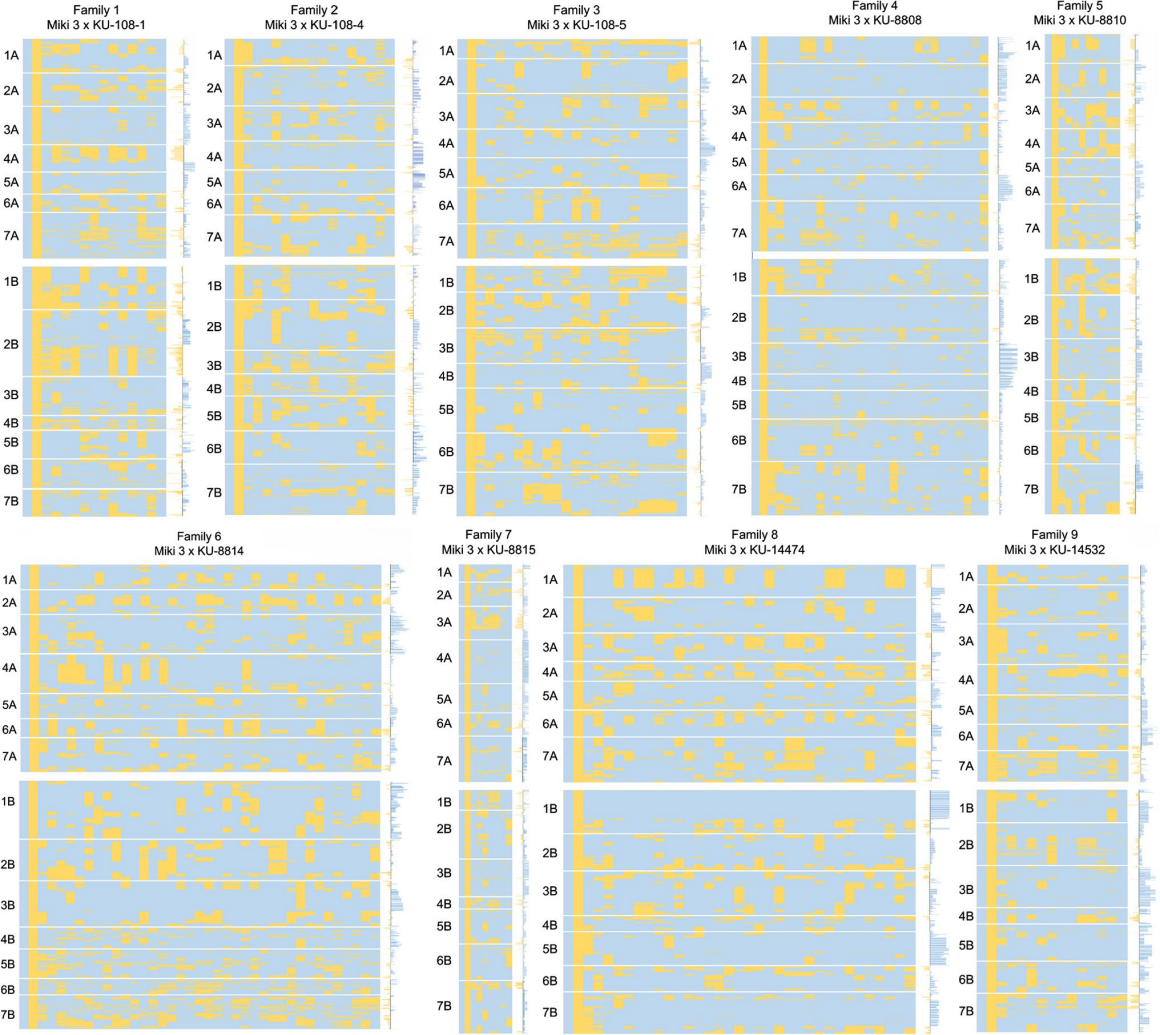
Graphical genotyping describes recombination between wild emmer wheat (WEW) and recurrent parent ‘Miki 3’ genomes in the nine multiple derivative line families. Conditional formatting in Microsoft Excel generated the plots from polymorphic markers between ‘Miki 3’ (pale blue) and WEW (yellow). Blue and yellow colors spread in each family indicate the ‘Miki 3’ and WEW genomes, respectively. Letter Codes on the left indicate chromosome numbers. The leftmost column in each family indicates the ‘Miki 3’ genome and the following columns indicate WEW genomes. Marker deviation from the expected Mendelian segregation ratio of 3:1 is plotted to the right of each family plot: blue, toward ‘Miki 3’; yellow, toward WEW donor parent. The middle blackline indicates no deviation from the expected 3:1 ratio (color figure online)

Following one backcross event, the expected frequencies of ‘Miki 3’ and WEW genomes in the MDLs are 75% ‘Miki 3’ and 25% WEW. The *χ*-test for each chromosome found a deviation from the expected ratio toward one or other parent (mainly the recurrent parent) on most of the chromosomes in both A and B genomes in all families (Fig. 4).

### Genetic diversity of the MDL population

**Fig. 5:**
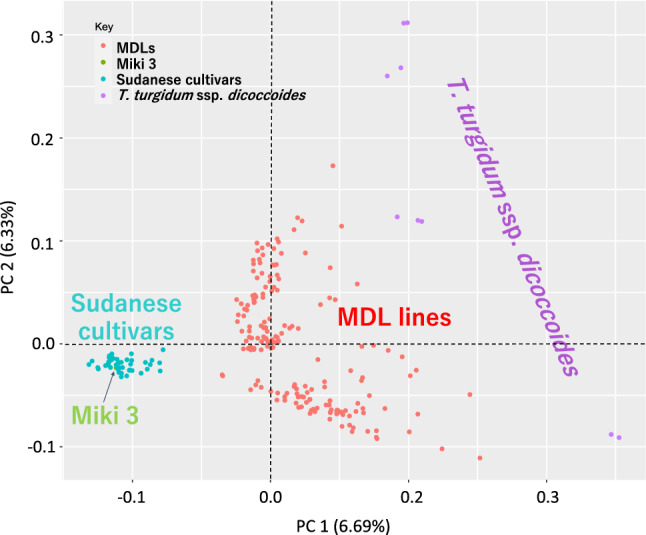
Principal component analysis (PCA) of diversity in multiple derivative lines, parental lines, and Sudanese cultivars based on 7 275 SNP markers (color figure online)

**Fig. 6:**
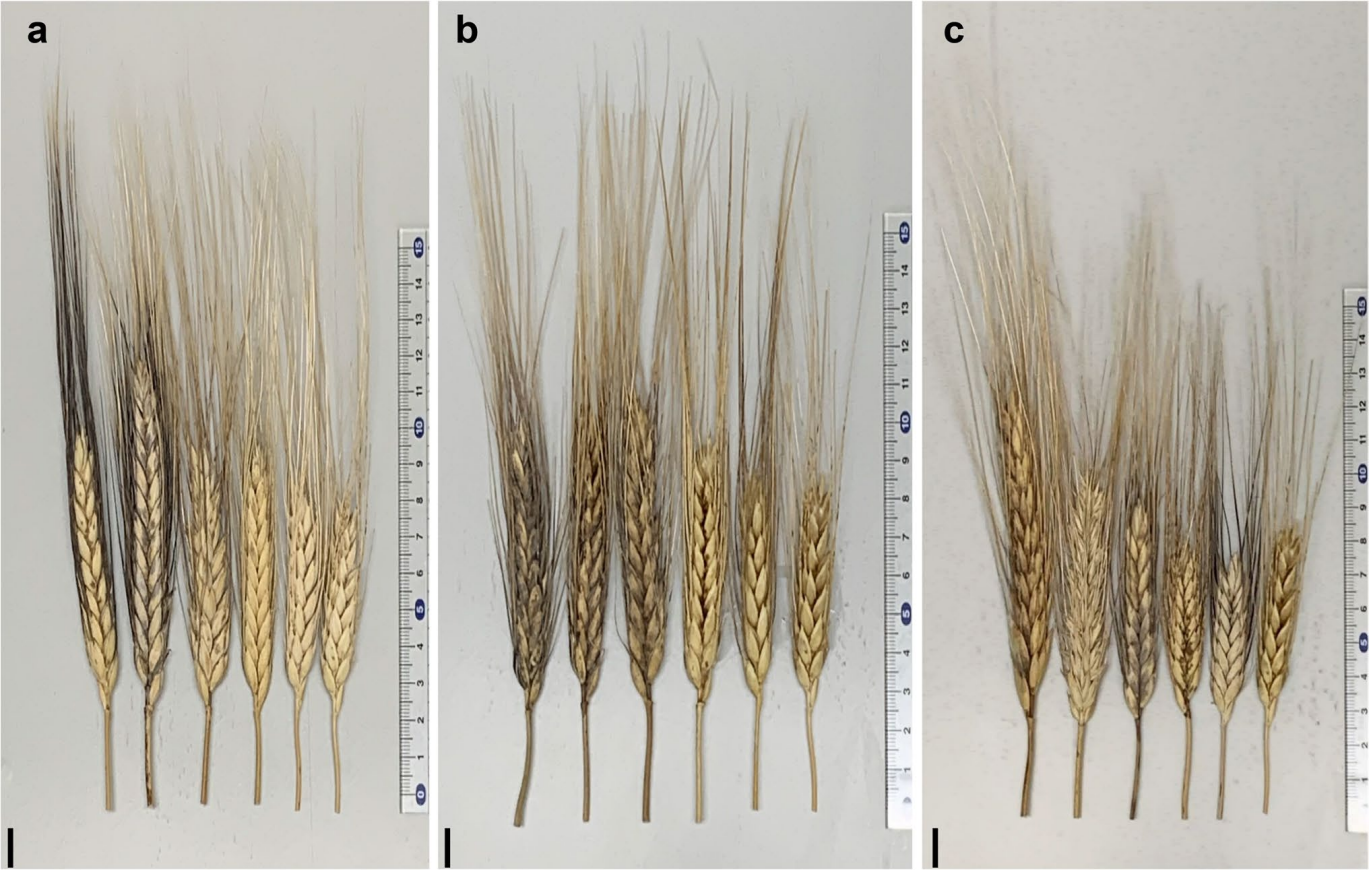
Diversity of shape among some multiple derivative lines: **a** variation of spike length, awn length, and color; **b** variation of glume color; **c** variation of spike shape. Scale bars, 1 cm (color figure online)

We performed PCA to estimate genetic diversity among the MDL lines, their parents, and the 43 Sudanese durum wheat cultivars using 7 275 SNPs (Fig. 5). The groups formed three clear clusters (Fig. 5). PCA divided the nine WEW accessions into two groups, with two and seven accessions, in agreement with the phylogenetic analysis. The Sudanese cultivars and ‘Miki 3’ were clustered together. However, the MDL lines were divided into two groups, one closer to the upper seven WEW accessions, the other closer to the lower two. The MDL lines were placed between the Sudanese group (including ‘Miki 3’) and the WEW accessions. The MDL lines explained much more genetic diversity than the Sudanese cultivars. However, the PCA showed low variance in principal components, PC 1 (6.69%) and PC 2 (6.33%) (Fig. 5), and 26 PCs were necessary to capture 50% of the molecular variance (Supplementary Fig. 2), suggesting limited structure in the population. Discriminant analysis of principal components (DAPC) showed nine genetic groups in the MDL population (Supplementary Fig. 3a). However, Bayesian information criterion (BIC) provided five main clusters (Supplementary Fig. 3b). The overlap between some families is consistent with the degree of similarity observed among the nine WEW accessions (Fig. 2). Although the PCA explained low variation, the MDL population had difference phenotypic variation in spike length, size and shape, awn color and length, and glume color (Fig. 6).

We used Nei’s gene diversity index and the polymorphic information content (PIC) to evaluate genetic diversity within the MDL population. Nei’s index indicates the probability that two randomly chosen alleles from a population are different (Xu and Vayena 2015). PIC values provide an estimate of the likelihood of finding polymorphism between two random samples of germplasm. Numbers of SNP and polymorphic markers, Nei’s genetic diversity index, and PIC values estimated for each chromosome and genome are listed in Table 2. Out of the 7 275 SNP markers, 2 093 were highly polymorphic across the MDLs. The Nei’s genetic diversity was 0.2476. The A genome had a genetic diversity of 0.2559 and a PIC of 0.2333; the B genome had a genetic diversity of 0.2384 and a PIC of 0.2182. The differences between A and B genomes in genetic diversity and PIC were not significant (paired *t*-test = 2.126, *P* = 0.0775, and paired *t*-test = 2.255, *P* = 0.0649, respectively).

**Table 2:** Total number of markers, number of polymorphic markers, Nei's genetic diversity index, and PIC in each chromosome in the 178 multiple derivative lines (MDLs)

Genome	No. of markers	No. of polymorphic markers	Nei's genetic diversity index	PIC^†^
*A genome*				
1A	407	145	0.2973	0.2685
2A	585	152	0.2269	0.2091
3A	485	173	0.2655	0.2411
4A	389	119	0.2636	0.2383
5A	577	159	0.2419	0.2220
6A	318	91	0.2418	0.2215
7A	552	172	0.2544	0.2329
Mean in A genome	473.3	144.4	0.2559	0.2333
*B genome*				
1B	525	153	0.2531	0.2315
2B	715	208	0.2259	0.2073
3B	604	182	0.2384	0.2206
4B	466	123	0.2369	0.2199
5B	635	135	0.2119	0.1906
6B	472	107	0.2275	0.2095
7B	545	174	0.2756	0.2481
Mean in B genome	566.0	154.6	0.2385	0.2182
Total / Mean	7275	2093	0.2476	0.2264

^†^PIC: Polymorphic information content

Differentiation between the MDLs and the nine WEW accessions was assessed by AMOVA based on PhiPT values, which found 9% of variance among and 91% within populations (Nm = 5.31, Phipt = 0.086), indicating a high gene exchange (low genetic differentiation) between the two groups (Table 3).

**Table 3:** Analysis of molecular variance between nine wild emmer wheat (WEW) accessions and 178 multiple derivative lines (MDLs) using 7275 SNPs markers

Source of variation	df^†^	Sum of squares	Mean of Squares	Estimate variance	Percentage of variance
Among population	1	1116.321	1116.321	10.194	9%
Within population	186	80,686.553	426.913	216.913	91%
Total	187	81,802.874		467.107	
Pairwise population (Phipt)	0.086				
Nm (haploid)	5.31				

^†^Degree of freedom

^†^Nm (haploid number of migrants)

### GWA analysis

To validate the usefulness of the MDL population for mapping traits and gene mining, we performed GWA analyses of DH and PTH in Dongola and Tottori.

**Fig. 7:**
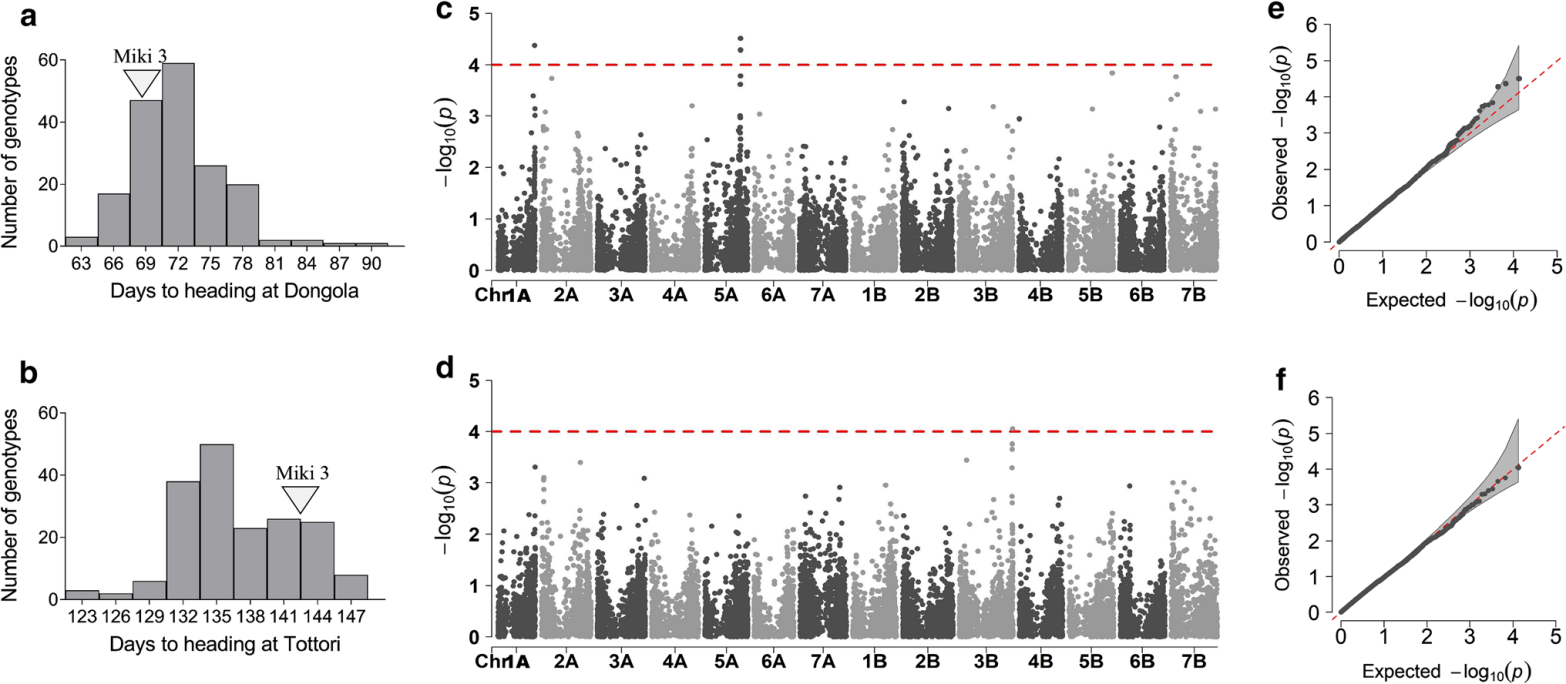
Genome-wide association analysis of days to heading at **a**, **c**, **e** Dongola and **b**, **d**, **f** Tottori in the multiple derivative line population: **a**, **b** frequency distribution; **c**, **d** Manhattan plots (dashed red line indicates significance threshold); **e**, **f**, quantile–quantile plots. 'Miki 3' is the backcross parent of MDLs (color figure online)

**Fig. 8:**
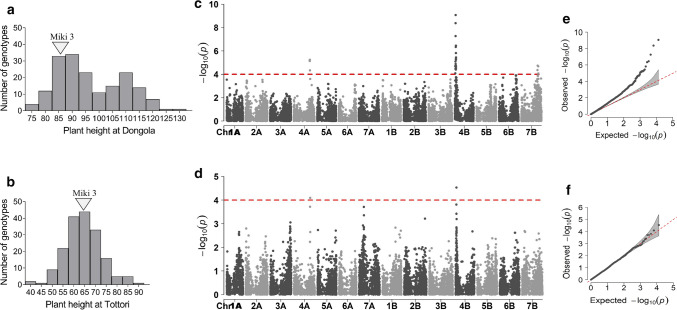
Genome-wide association analysis of plant height at **a**, **c**, **e** Dongola and **b**, **d**, **f** Tottori in the multiple derivative line population: **a**, **b** frequency distribution; **c**, **d** Manhattan plots (dashed red line indicates significance threshold); **e**, **f**, quantile–quantile plots. 'Miki 3' is the backcross parent of MDLs (color figure online)

**Fig. 9:**
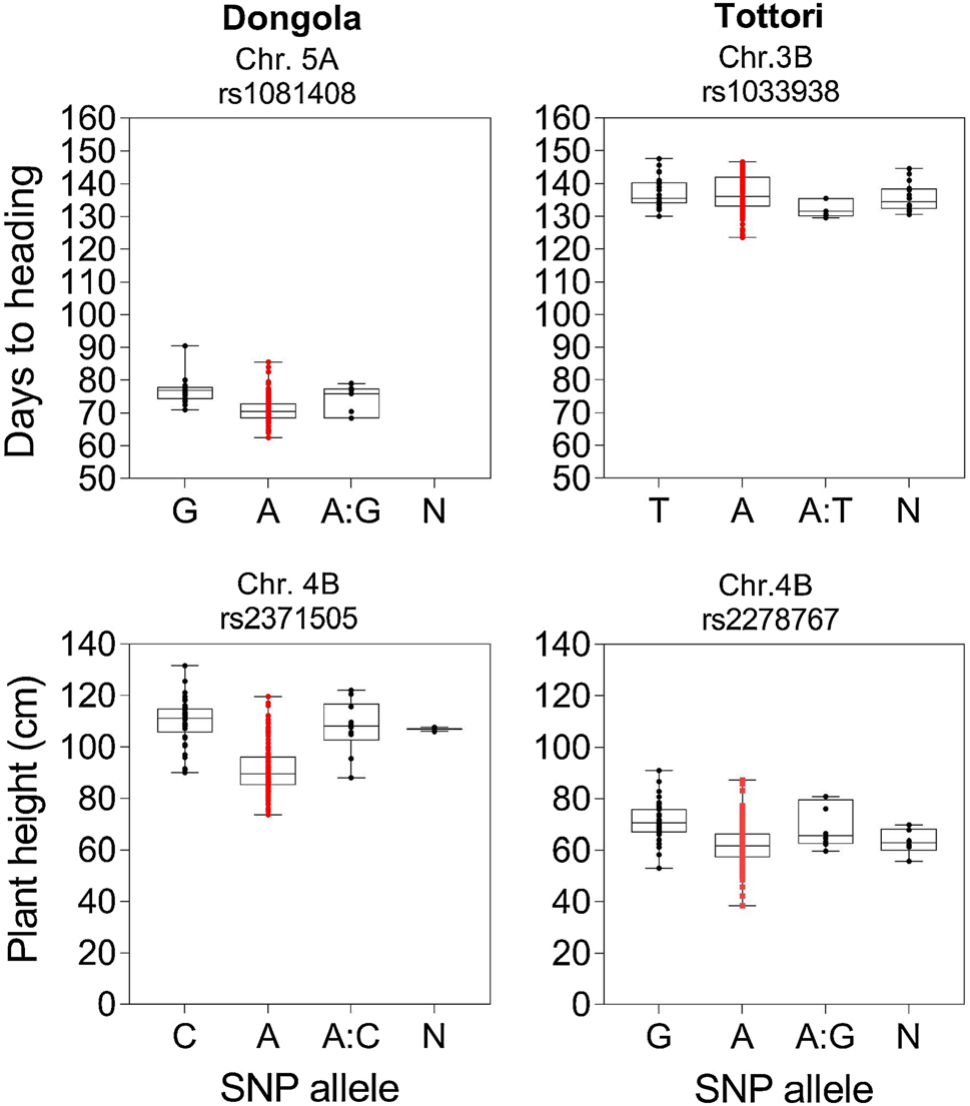
Effect of marker-trait associations on days to heading and plant height in MDL population evaluated in Dongola or Tottori. A, adenine; C, cytosine; G, guanine; T, thymine; N, unknown. Red dots are the allele of ‘Miki 3’ (color figure online)

We found highly significant differences (*P* ≤ 0.001) among the MDL lines for both traits. DH had a wide range of variation, from 62 to 90 days in Dongola (Fig. 7a) and 123 to 147 days in Tottori (Fig. 7b). PHT ranged from 73.5 to 131.5 cm in Dongola and 38.3 to 90.9 cm in Tottori (Fig. 8a, b). GWA analysis identified three significant marker-trait associations (MTAs) for DH in Dongola, on Chrs 1A (1 MTA) and 5A (2 MTAs) (Fig. 7c). These MTAs explained 13.8 to 14.3% of the genetic variance (Table 4). In Tottori, GWA analysis revealed one significant MTA for DH, on Chr 3B (Fig. 7d), which explained 13.9% of the genotypic variance. GWAS detected 28 significant MTAs for PHT at three genomic regions: 26 MTAs in Dongola on Chrs 4A, 4B, and 7B; and 2 MTAs in Tottori on Chrs 4A and 4B (Fig. 8c, d; Table 4). The MTAs explained 13.7 to 32.0% of the genetic variance in Dongola and 10.8 to 14.9% in Tottori. We identified two stable markers 2252536 on Chr 4A and 2278767 on Chr 4B associated with PHT in both locations (Table 4). Alleles contributing to early heading and short PHT were found to be derived from the recurrent parent ‘Miki 3’ in both locations (Fig. 9).

**Table 4:** Marker–trait associations of days to heading (DH) and plant height (PHT) in multiple derivative lines (MDLs) grown under two environments, Dongola, and Tottori

Chromosome	Marker	Position	Trait	Environment	*P-*value	Marker *R*^2^
1A	2,262,791	5.77E+08	DH	Dongola	4.27 × 10^−5^	0.142
3B	1,033,938	8.29E+08	DH	Tottori	8.90 × 10^−5^	0.139
4A	2,252,536	5.72E+08	PHT	Dongola	5.80 × 10^−6^	0.146
		5.72E+08		Tottori	8.01 × 10^−5^	0.108
	1.3E+07	5.67E+08		Dongola	6.31 × 10^−6^	0.172
	1,087,149	5.7E+08		Dongola	7.44 × 10^−6^	0.143
	1,220,382	5.75E+08		Dongola	4.67 × 10^−5^	0.143
4B	2,371,505	29,297,345	PHT	Dongola	8.55 × 10^−10^	0.321
	1,216,462	24,384,093		Dongola	4.24 × 10^−9^	0.306
	1,064,354	26,614,161		Dongola	5.47 × 10^−8^	0.259
	1,863,400	38,841,165		Dongola	3.52 × 10^−7^	0.218
	2,278,767	30,576,288		Dongola	4.55 × 10^−7^	0.220
		30,576,288		Tottori	2.92 × 10^−5^	0.149
	991,096	38,841,231		Dongola	1.53 × 10^−6^	0.197
	1,300,855	39,949,802		Dongola	3.65 × 10^−6^	0.180
	2,283,875	37,707,737		Dongola	4.80 × 10^−6^	0.176
	1,088,389	39,319,967		Dongola	5.76 × 10^−6^	0.173
	1,212,987	21,575,326		Dongola	8.14 × 10^−6^	0.168
	5.5E+07	28,796,166		Dongola	1.32 × 10^−5^	0.163
	1,003,062	38,154,454		Dongola	1.91 × 10^−5^	0.159
	1,092,216	12,250,336		Dongola	2.01 × 10^−5^	0.154
	4,010,028	36,999,359		Dongola	2.43 × 10^−5^	0.152
	1,091,494	39,054,980		Dongola	3.14 × 10^−5^	0.156
	984,917	21,287,734		Dongola	3.50 × 10^−5^	0.147
	3,958,247	35,840,364		Dongola	4.04 × 10^−5^	0.147
	1,101,888	17,488,180		Dongola	8.42 × 10^−5^	0.133
5A	1,081,408	5.58E+08	DH	Dongola	3.11 × 10^−5^	0.143
	1,074,046	5.58E+08			5.22 × 10^−5^	0.138
7B	1,017,437	5.85E+08	PHT	Dongola	1.85 × 10^−5^	0.168
	1,214,796	6.1E+08			2.12 × 10^−5^	0.160
	1,058,067	5.82E+08			4.45 × 10^−5^	0.142
	2,257,383	6.11E+08			8.56 × 10^−5^	0.137

*R*^2^ Genetic variance explained by markers

## Discussion

Although durum wheat was domesticated about 10,000 years ago (Shewry 2009), the official breeding program does not exceed 120 years (Taranto et al. 2020). A robust genetic bottleneck occurred during this gap as the domestication process caused substantial genetic erosion (Maccaferri et al. 2019). However, to meet the needs of a growing human population and the increasing climate change scenario, crop production would need to be further improved especially through the use of genetic resources of wild progenitors to introgress agronomically superior and adaptive traits.

We developed a new population of multiple derivative lines (MDLs) that harbor fragments of wild emmer wheat (WEW) diversity in its gene pool. This study elucidated the genetic potential of this population by identifying novel traits and MTAs from the wild relative progenitor *T. turgidum* ssp*. Dicoccoides,* and the suitability of this population for wheat breeding.

The WEW accessions from Iraq clustered separately from those originating from Israel (Fig. 2). Two WEW lineages exist in its distribution area: the western lineage, found in Jordan, Syria, Lebanon, and Israel, and the central-eastern lineage, found in Turkey, Iran, and Iraq (Mori 2003; Ozkan et al. 2005; Matsuoka 2011; Peng et al. 2011). As the nine WEW accessions used in our study represent the western lineage (Israel) and the central-eastern lineage (Iraq), we speculate that they cover the spectrum of diversity present in WEW, although the number is limited.

Although MDL is a mixed population, we could identify the pedigree of each of the 178 lines using DArTseq markers. Such analyses allows us to track the origin of useful traits and use the corresponding accessions for further crossing in the breeding program. Although the MDL population was created by mixing an equal number of seeds from each cross, the nine WEW accessions contributed different numbers of individuals among the lines (Fig. 3). We attributed this imbalance to both natural and artificial selection during the production of the MDL population, which is in agreement with a previous study in bread wheat by Gorafi et al. (2018).

The MDL population (BC_1_F_6_) has an expected contribution of 75% from ‘Miki 3’ and 25% from the donor WEW accessions. All MDLs showed a deviation from the expected ratio toward one or the other parent, especially the recurrent parent (Fig. 4). We attributed this deviation to the fact that the 178 accessions used here were selected for good agronomic performance under the Sudanese environment. This selection removed all individuals with unsuitable WEW traits such as brittle rachis, glume tenacity, and non-free-threshing type, and consequently reduced the contribution of WEW alleles. The chromosomes within families that showed deviation toward one parent could be a result of competition between gametes for preferential fertilization or from gamete or zygote abortion. The number of individuals within each family was low, ranging from 7 to 37 (Fig. 3). Therefore, distortion could be due to non-biological factors derived from low population size and genotyping errors (Alheit et al. 2011).

Phylogenetic analysis showed a difference between the nine WEW accessions and modern Sudanese durum wheat cultivars including ‘Miki 3’ (Fig. 2). This result revealed the loss of genetic diversity in the Sudanese cultivars caused by domestication and breeding (Maccaferri et al. 2019). The PCA placed the MDLs between the WEW accessions and modern cultivars (Fig. 5). The MDL families grouped by DAPC analysis (Supplementary Fig. 3a) reflect the genetic makeup of the nine WEW. Although the DAPC analysis showed the nine genetic groups, the Bayesian information criterion (BIC) revealed five clusters in the MDLs (Supplementary Fig. 3b). This result could be due to the high similarity among some WEW accessions. For instance, accessions KU-8814 and KU-8815 were derived from the same line, and their progenies (families six and seven) are highly overlapped (Supplementary Fig. 3a). Interestingly, the DAPC grouping seems to reflect the geographical origin of the nine WEW, in agreement with phylogenetic analysis (Fig. 2). These results revealed that the MDLs provide an effective platform with which to harness the WEW diversity.

We evaluated days to heading and plant height in Dongola and Tottori to validate the suitability of the MDL population for MTA identification and to dissect the WEW genes. GWA analysis identified two genomic loci on Chrs 1A and 5A with relevant DH effects in Dongola (Fig. 7c). Flowering time of wheat is controlled by a network of genes integrating major vernalization genes located on Chrs 5A (*Vrn-1* and *Vrn-2*) and 7BS (*Vrn-3*); a series of homoeologous photoperiod response genes on group 2 chromosomes; and earliness genes on Chrs 1A, 3A, and 3B (Pánková et al. 2008; Fowler et al. 2016). The significant MTAs for DH identified in Dongola are located on the chromosomes reported to harbor major genes associated with DH. GWA analysis for DH showed one MTA on Chr 3B (Fig. 7d) positioned at the earliness per se locus detected in Tottori (Pánková et al. 2008). Kobayashi et al. (2016) evaluated 96 Japanese wheat cultivars (JWC) for DH in autumn and spring sowing and found significant MTAs on Chr 3B associated with DH in autumn sowing. The differences in the GWA results between Dongola and Tottori arose from the different climatic conditions during the period of evaluation. Although Dongola is regarded as a relatively cooler location than other places in Sudan, it is warmer than Tottori, and this difference may explain the detection of the vernalization loci in Dongola. Distelfeld et al. (2009) reported that *Vrn-1* genes regulate the transition from vegetative to reproductive phase in response to temperature and thus determine the spring and winter growth habit. Therefore, evaluation of DH in the MDL population revealed the three genomic loci reported to control flowering time in wheat (Pánková et al. 2008; Kobayashi et al. 2016; Gupta et al. 2020).

GWA analysis for PHT revealed two common genomic loci in Dongola and Tottori on Chrs 4A and 4B (Fig. 8c, d). These MTAs correspond to *Reduced height* alleles *Rht-A1* and *Rht-B1* (Wilhelm et al. 2013). The introduction of *Rht-1* in the 1960s during the Green Revolution led to improved lodging resistance and yield. Similar results of GWA analysis for PHT between Dongola and Tottori indicated that the specific environment did not restrict the occurrence of *Rht-1*. On the other hand, the MTAs detected on Chr 7B that coincided with the location of the *Rht13* allele appeared only in Dongola (Fig. 8c, Ellis et al. 2005).

Most of the earlier and shorter genotypes contain alleles derived from the ‘Miki 3’ (Fig. 9). Bentley et al. (2011) reported that mutations associated with the early heading phenotype are absent from wild tetraploid wheat, but were predominate on chromosome 2A in modern durum wheat, suggesting that they originated after domestication and were selected for the improvement of adaptation. Also, more than 70% of the modern wheat cultivars incorporate one of the original semi-dwarfing genes defining the characteristics of the ‘Green Revolution’ (Jobson et al. 2019).

A significant advantage for plant geneticists comes from creating diverse experimental populations that enable the genetic dissection of complex traits to support plant breeding. To this end, Gorafi et al. (2018) proposed an efficient platform in bread wheat named multiple synthetic derivative (MSD) lines that possess a large diversity of *Aegilops tauschii* in a modern bread wheat cultivar. This method facilitates the exploration of the diversity of wild wheat progenitors in one population. Our platform is similar to that of the MSDs: the MSD harnesses the diversity of *Ae. tauschii* (the D-genome donor of hexaploid wheat), and the MDL platform exploits the diversity of WEW A and B genomes. Moreover, compared with multi-parental advanced-generation inter-cross and nested association-mapping strategies, the MDL/MSD platforms allow us to save time by starting evaluation and selection of desired phenotypes at an early generation. Combining new advances in speed-breeding methods (Hickey et al. 2019; Wanga et al. 2021), the MDL/MSD strategy could offer a rapid way to utilize the diversity of wild relatives for wheat improvement.

The MDL lines are being tested under heat stress conditions in Sudan to further evaluate the MDLs potential. Our preliminary findings showed several potential heat-tolerant lines with good agronomical performance (data not shown). We believe that the MDL platform could provide valuable materials for different breeding purposes such as drought tolerance, salinity tolerance, and end-use quality improvements just as the MSD population of bread wheat is a useful source of heat and drought tolerance (Elbashir et al. 2017; Itam et al. 2020; Elhadi et al. 2021). The uniform genetic backgrounds of these platforms allow accurate evaluation of quantitative traits of wild species (*Ae. tauschii* or WEW) as traits of cultivated wheat species (*T. aestivum* or *T. turgidum* ssp. *durum*). Furthermore, diversity in the MDL has a potential to improve the diversity of A and B genomes of bread wheat. Efforts are currently underway to accumulate these platforms' diversity by intercrossing selected MDL/MSD lines and developing lines with traits linked to heat and combined heat-drought stress tolerance.

## Conclusion

Our results revealed that multiple derivative lines offer a promising tool to harness the diversity in wild emmer wheat in a short time without a large investment. The GWA results showed the suitability of the MDLs for the identification of marker–trait associations. Thus, the MDL population is an attractive resource with which to uncover the genes of wild emmer wheat and facilitate their use for bread and durum wheats improvement.

## Supplementary Information

Below is the link to the electronic supplementary material.

Supplementary file1 (PPTX 38 kb)

Supplementary file2 (PPTX 76 kb)

Supplementary file3 (PPTX 187 kb)

## Data Availability

All the data and materials in this study are available through the Laboratory of Arid Land Plant Resources, Tottori University (http://www.alrc.tottori-u.ac.jp/plant/total_introduction.html).
