# Supplementary material for: Harnessing the diversity of wild emmer wheat for genetic improvement of durum wheat
Source: Theor Appl Genet. 2022 Mar 7;135(5):1671–84. doi: 10.1007/s00122-022-04062-7 (PMC9110450; doi:10.1007/s00122-022-04062-7)
Supplement: Supplementary file 1 — Supplementary file1 (PPTX 38 kb) [file 122_2022_4062_MOESM1_ESM.pptx]

## Slide 1
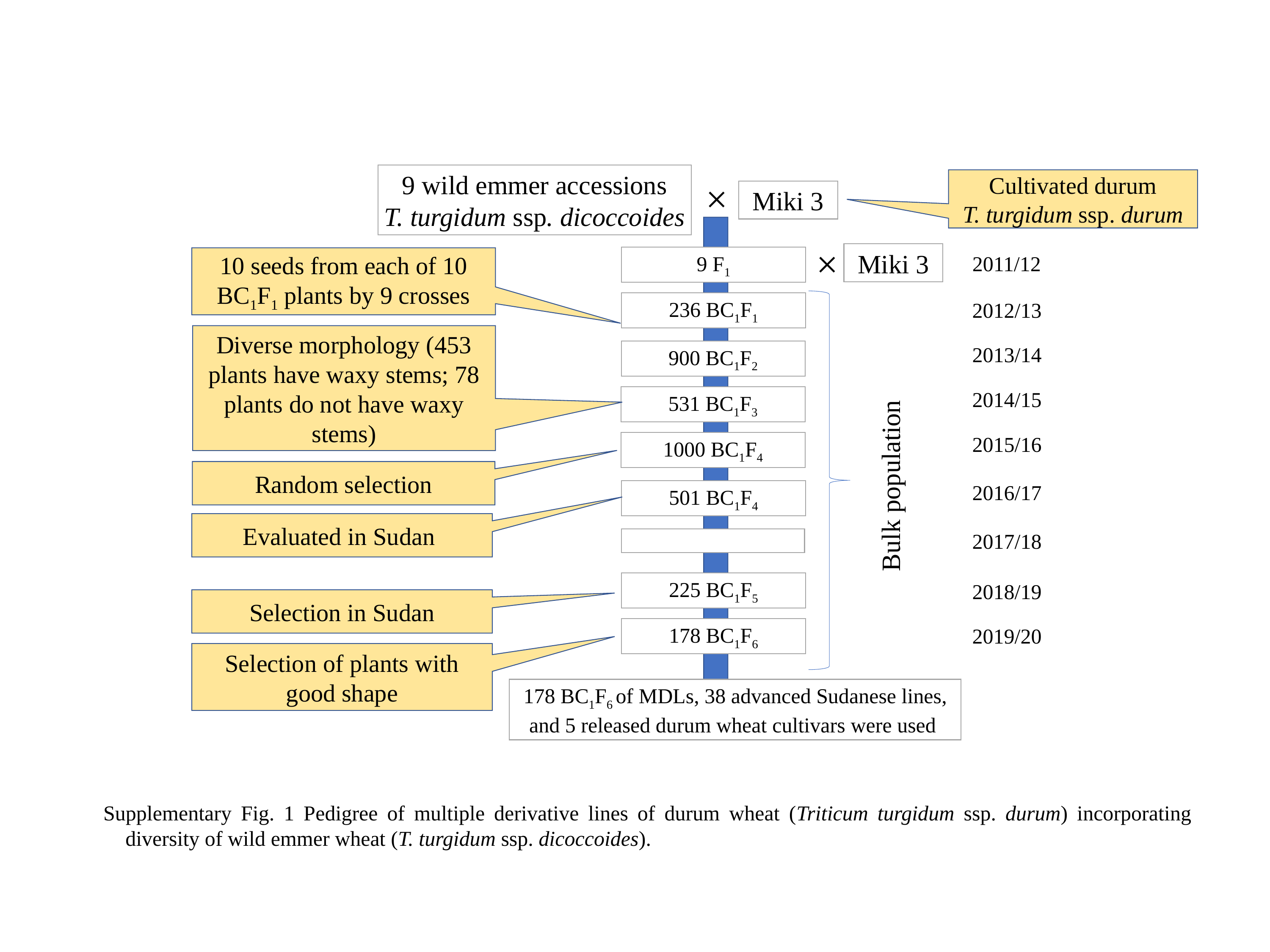

9 wild emmer accessions
T. turgidum ssp. dicoccoides
Cultivated durum
T. turgidum ssp. durum
×
Miki 3
×
Miki 3
2011/12
9 F1
10 seeds from each of 10 BC1F1 plants by 9 crosses
236 BC1F1
2012/13
Diverse morphology (453 plants have waxy stems; 78 plants do not have waxy stems)
2013/14
900 BC1F2
2014/15
531 BC1F3
2015/16
1000 BC1F4
Random selection
Bulk population
2016/17
501 BC1F4
Evaluated in Sudan
2017/18
225 BC1F5
2018/19
Selection in Sudan
178 BC1F6
2019/20
Selection of plants with good shape
178 BC1F6 of MDLs, 38 advanced Sudanese lines, and 5 released durum wheat cultivars were used
Supplementary Fig. 1 Pedigree of multiple derivative lines of durum wheat (Triticum turgidum ssp. durum) incorporating diversity of wild emmer wheat (T. turgidum ssp. dicoccoides).
