# Supplementary material for: Harnessing the diversity of wild emmer wheat for genetic improvement of durum wheat
Source: Theor Appl Genet. 2022 Mar 7;135(5):1671–84. doi: 10.1007/s00122-022-04062-7 (PMC9110450; doi:10.1007/s00122-022-04062-7)
Supplement: Supplementary file 2 — Supplementary file2 (PPTX 76 kb) [file 122_2022_4062_MOESM2_ESM.pptx]

## Slide 1
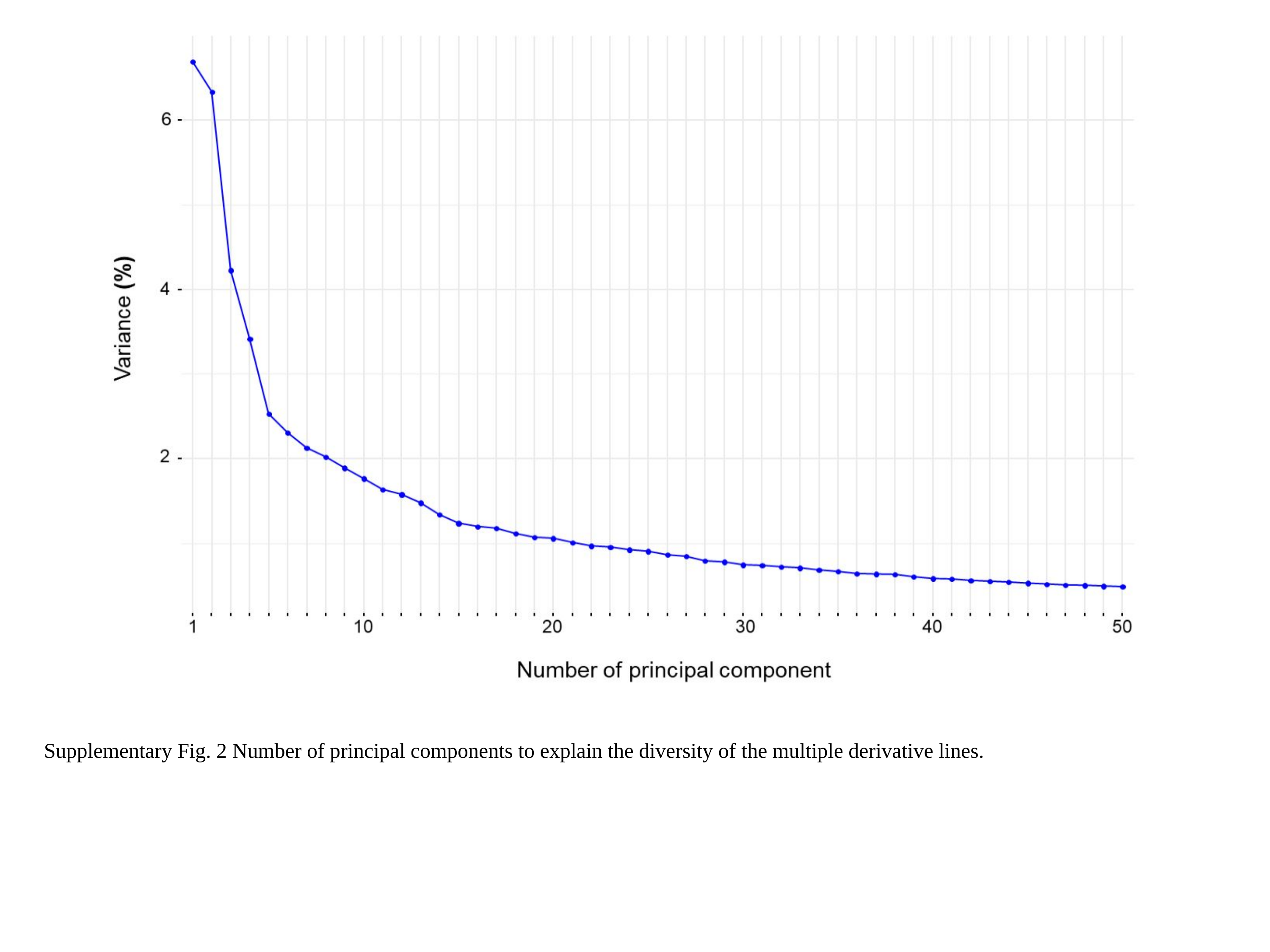

Supplementary Fig. 2 Number of principal components to explain the diversity of the multiple derivative lines.
