# Supplementary material for: Harnessing the diversity of wild emmer wheat for genetic improvement of durum wheat
Source: Theor Appl Genet. 2022 Mar 7;135(5):1671–84. doi: 10.1007/s00122-022-04062-7 (PMC9110450; doi:10.1007/s00122-022-04062-7)
Supplement: Supplementary file 3 — Supplementary file3 (PPTX 187 kb) [file 122_2022_4062_MOESM3_ESM.pptx]

## Slide 1
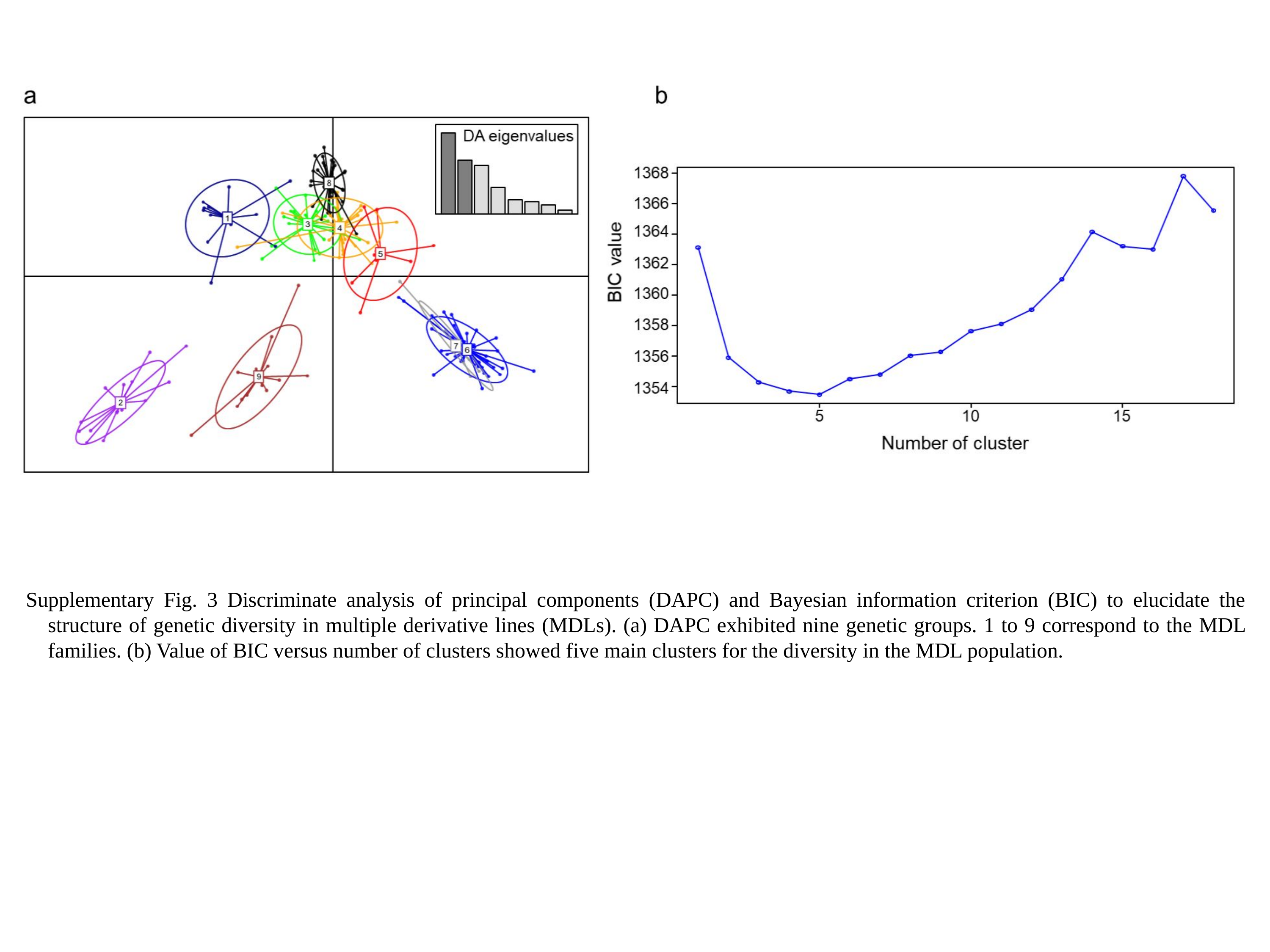

Supplementary Fig. 3 Discriminate analysis of principal components (DAPC) and Bayesian information criterion (BIC) to elucidate the structure of genetic diversity in multiple derivative lines (MDLs). (a) DAPC exhibited nine genetic groups. 1 to 9 correspond to the MDL families. (b) Value of BIC versus number of clusters showed five main clusters for the diversity in the MDL population.
